# Computing *F*-index, coindex and Zagreb polynomials of the *k*th generalized transformation graphs

**DOI:** 10.1016/j.heliyon.2020.e05781

**Published:** 2020-12-22

**Authors:** Durbar Maji, Ganesh Ghorai

**Affiliations:** Department of Applied Mathematics with Oceanology and Computer Programming, Vidyasagar University, Midnapore - 721102, India

**Keywords:** Mathematics, Molecular graph, Zagreb indices, Transformation graphs, *k*th generalized transformation graph, Zagreb polynomial and co-polynomial, *F*-index and its coindex

## Abstract

In chemical graph theory, forgotten topological index or *F*-index plays a crucial role to collect information about the properties of chemical compounds. The *k*th generalized transformation graphs of a molecular graph preserve the entire information on the molecular topology contained in the relevant molecular graph. In this paper, some exact expressions of the F-index and its co-index for the *k*th generalized transformation graphs are obtained. Also, the Zagreb polynomials, Zagreb co-polynomials and their complements are computed.

## 1 Introduction

In theoretical chemistry, chemical compounds are often used to model different molecular structures which are graphically represented as molecular graphs in which atoms as nodes and chemical bonds as edges. Throughout this paper, we consider only molecular graphs that are simple. Let X=(n,m) be such a graph of order *n* and size *m* with vertex set V(X) and edge set E(X) such that |V(X)|=n and |E(X)|=m. Also, for any vertex p∈V(X), d(p/X) denotes the degree of that vertex in *X*. The graph $\bar{X}$ be the complement of *X* with the same vertex set V(X) and for any edge, $pq\in E(\bar{X})$ exists if and only if pq∉E(X). So, it is clear that $E(X)\cup E(\bar{X})=E(K_{n})$ and $\left|E(\bar{X})|=\left(\begin{array}{c} \left|V(X)\right|\\ 2 \end{array}\right)-|E(X)\right|$. Therefore, the degree of $p\in V(\bar{X})$ is given by $d(p/\bar{X})=|V(X)|-1-d(p/X)$.

We will follow reference [1] for undefined notation and terminology. Graph theory [2] provides a link between mathematics and chemistry by an important tool named topological index. Forty eight years ago the two oldest graph invariants namely first and second Zagreb indices are introduced by Gutman and Trinajestic [3]. The first Zagreb index is denoted as $M_{1}(X)$ and is defined by $M_{1}(X)=\sum_{p\in V(X)}d^{2}(p/X)=\sum_{pq\in E(X)}[d(p/X)+d(q/X)]$. Doslic [4] defined the first Zagreb coindex as $\bar{M_{1}}(X)=\sum_{pq\notin E(X)}[d(p/X)+d(q/X)]$. Analogously to another topological index in [3] is calculated as the sum of the power three of degrees of the vertices of a graph. It is further found in Furtula et al. in [5]. This index is named as forgotten topological index or shortly, *F*-index which also influences the total *π*-electron energy (*ϵ*). This index is symbolically expressed as $F(X)=\sum_{p\in V(X)}d^{3}(p/X)=\sum_{pq\in E(X)}[d^{2}(p/X)+d^{2}(q/X)]$. In 2016, De [6] introduced a new graph invariant, the *F*-coindex as follows $\bar{F}(X)=\sum_{pq\in E(\bar{X})}(d^{2}(p/X)+d^{2}(q/X))$. Transformation graphs preserve the entire information from the original graph into new transformed structure. For details see [7]. The basic properties of transformation graphs can be followed in [8]. For more on transformation graphs refer to [9], [10]. We refer our readers to [11] for recent study.

Analogous to the Zagreb indices, Fath-Tabar [12] defined the first, second and third Zagreb polynomials as

$$M_{1}(X,x)=\sum_{pq\in E(X)}x^{d(p/X)+d(q/X)}$$

$$M_{2}(X,x)=\sum_{pq\in E(X)}x^{d(p/X)d(q/X)}$$

and

$$M_{3}(X,x)=\sum_{pq\in E(X)}x^{\left|d(p/X)-d(q/X)\right|}$$

respectively.

On the base of Zagreb coindices, Basavanagoud and Jakkannavar [13] defined three new graph polynomials, namely the first, second and third Zagreb co-polynomials. They are as follows

$$\bar{M_{1}}(X,x)=\sum_{pq\notin E(X)}x^{d(p/X)+d(q/X)}$$

$$\bar{M_{2}}(X,x)=\sum_{pq\notin E(X)}x^{d(p/X)d(q/X)}$$

and

$$\bar{M_{3}}(X,x)=\sum_{pq\notin E(X)}x^{\left|d(p/X)-d(q/X)\right|}$$

respectively.

Additionally, Shuxian [14] presented two new polynomials related to the first Zagreb index like as

$$M_{1}^{⁎}(X,x)=\sum_{p\in V(X)}d(p/X)x^{d(p/X)}$$

and

$$M_{0}(X,x)=\sum_{p\in V(X)}x^{d(p/X)}.$$

Further, Bindusree et al. [15] introduced the following polynomials

$$M_{a,b}(X,x)=\sum_{pq\in E(X)}x^{ad(p/X)+bd(q/X)}$$

and

$$M_{a,b}^{\prime }(X,x)=\sum_{pq\in E(X)}x^{\left(d(p/X)+a)(d(q/X)+b\right)}.$$

Similarly, Basavanagoud et al. [13] also defined

$${\bar{M}}_{a,b}(X,x)=\sum_{pq\notin E(X)}x^{ad(p/X)+bd(q/X)}$$

and

$${\bar{M^{\prime }}}_{a,b}(X,x)=\sum_{pq\notin E(X)}x^{\left(d(p/X)+a)(d(q/X)+b\right)},$$

where *x* is to be chosen as a variable.

For different recent study of *F*-index and its co-index, we refer to [8], [16], [17]. In order to know more about Zagreb polynomials and its co-polynomials for various transformation graphs see [15], [18], [19]. The following Propositions are instrumental in proving for the present considerations.

Proposition 1 *[20]* *Let X be a graph with n vertices and m edges and* $\bar{X}$ *be the complement of X. Then*

(i) $M_{1}(\bar{X})=n{\left(n-1\right)}^{2}-4m(n-1)+M_{1}(X)$*.*
(ii) $\bar{M_{1}}(X)=\bar{M_{1}}(\bar{X})=2m(n-1)-M_{1}(X)$*.*
(iii) $\bar{M_{2}}(X)=2m^{2}-\frac{1}{2}M_{1}(X)-M_{2}(X)=\bar{m}{\left(n-1\right)}^{2}+M_{2}(\bar{X})-(n-1)M_{1}(\bar{X})$*.*
(iv) $\bar{M_{2}}(\bar{X})=m{\left(n-1\right)}^{2}-(n-1)M_{1}(X)+\bar{M_{2}}(X)$*.*

Proposition 2 *[21]* *Let X be an* (n,m) *graph. Then*

(i) $M_{1}(X_{k}^{++})={\left(k+1\right)}^{2}M_{1}(X)+4mk$*.*
(ii) $M_{1}(X_{k}^{+-})=m^{2}k(nk+4-4k)+{\left(k-1\right)}^{2}M_{1}(X)+{\left(n-2\right)}^{2}km$*.*
(iii) $M_{1}(X_{k}^{-+})=4m(n-1)(k-1)+{\left(k-1\right)}^{2}M_{1}(X)+n{\left(n-1\right)}^{2}+4mk$*.*
(iv) $M_{1}(X_{k}^{--})=n{\left(n+km-1\right)}^{2}-4m(k+1)(n+km-1)+{\left(k+1\right)}^{2}M_{1}(X)+km{\left(n-2\right)}^{2}$*.*

Proposition 3 *[6]* *Let X be a graph of order n and size m. Then*

(i) $F(\bar{X})=n{\left(n-1\right)}^{3}-6m{\left(n-1\right)}^{2}+3(n-1)M_{1}(X)-F(X)$*.*
(ii) $\bar{F}(X)=(n-1)M_{1}(X)-F(X)=2\bar{m}{\left(n-1\right)}^{2}+F(\bar{X})-2(n-1)M_{1}(\bar{X})$*.*
(iii) $\bar{F}(\bar{X})=2m{\left(n-1\right)}^{2}-(n-1)M_{1}(X)-\bar{F}(X)$*.*

## 2 The *k*th generalized transformation graphs $X_{k}^{uv}$

The *k*th generalized transformations which was introduced by Jummannaver et al. [21] is the new graphical transformations of generalized transformations [18], [22] of a graph. This concept was developed by using semitotal-point graph which was defined by Sampathkumar and Chikkodimath in [23] and later put forward as the *k*th semitotal-point graph which was introduced by Jog in [24] of a graph.

Definition 1 [21] Consider X(V(X),E(X)) be a simple, connected graph and u,v are the two graph parameters having values + or -. The *k*-th generalized transformation graph (GTG) $X_{k}^{uv}$, is a new graph having $V(X_{k}^{uv})=V(X)\cup (E^{\prime }=\cup_{j=i}^{k}E_{j})$ as vertex set and $p,q\in V(X_{k}^{uv})$ such that the vertices *p* and *q* are adjacent in $X_{k}^{uv}$ if and only if the following conditions (i) and (ii) hold:

1. *p* and *q* are adjacent in *X* if u=+ and non adjacent in *X* if u=−.
2. p∈V(X) and $q\in E_{j}$, for some j∈β (where β=1,2,...,mor1(0)m). Suppose $e_{j}$ be the edge of *X* and $E_{1}$, $E_{2}$, ..., $E_{m}$ be the distinct edge set and each $E_{j}$ is corresponding to the edge $e_{j}$ in *X* such that $|E_{j}|=k,j=1(0)m$. The vertex *p* and $e_{j}$ are incident in *X* if v=+ and are not incident in *X* if v=−.

There exist 4-distinguished 2-permutations of {+,−}. The four graphical transformations in *X* like as $X_{k}^{++},X_{k}^{+-},X_{k}^{-+}$ and $X_{k}^{--}$ can be established. Also their complements like $\bar{X_{k}^{++}},\bar{X_{k}^{+-}},\bar{X_{k}^{-+}}$ and $\bar{X_{k}^{--}}$ can be obtained. The vertex *p* of $X_{k}^{uv}$ corresponding to a vertex *p* of *X* is referred to as a point vertex. Also, the vertex *e* of $X_{k}^{uv}$ corresponding to an edge *e* of *X* is referred to as a line vertex. To know more about the transformation graphs we can study in [25], [26].

**Figure 1:**
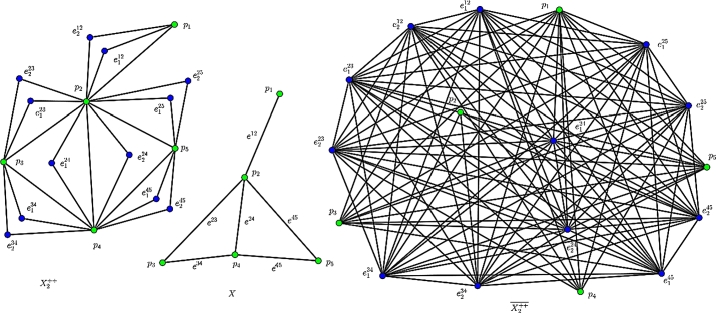
An example of $X_{k}^{++}$: $X_{2}^{++}$ of a graph *X*(5,6) and its complement $\bar{X_{2}^{++}}$.

There are two different types of edge partitions in the *k*th GTG $\left(X_{k}^{uv}\right)$ and its complement $\bar{X_{k}^{uv}}$. Firstly in each $X_{k}^{uv}$, the edges can be split into two parts like $E_{1}^{uv/k}$ and $E_{2}^{uv/k}$. Other hand, in each $\bar{X_{k}^{uv}}$, the partition of the edge set $E(\bar{X_{k}^{uv}})$ can be separated into three subsets like $E_{1}^{\bar{uv/k}}$, $E_{2}^{\bar{uv/k}}$ and $E_{3}^{\bar{uv/k}}$. As an example, the edges of $E(X_{k}^{++})$ can be split into two parts like $E_{1}^{++/k}=\{pq\in E(X)\}$ and $E_{2}^{++/k}=\{pe|\;\text{the vertex}\;\text{p}\;\text{and edge}\;\text{e}\;\text{in}\;\text{X}\;\text{are incident to each other}\}$ and the edges of $E(\bar{X_{k}^{++}})$ can be divided into three parts like $\bar{E_{1}^{++/k}}=\{pq\notin E(X)\}$, $\bar{E_{2}^{++/k}}=\{pe|\;\text{the}$ vertex *p* and edge *e* in *X* are not incident to each other} and $\bar{E_{3}^{++/k}}=\{ef|e,f\in E(X)\}$ (see Fig. 1).

## 3 The results on the F-index and its co-index of $X_{k}^{uv}$

The following theorems can be used to compute the results on *F*-index and its co-index of the *k*th GTG $X_{k}^{uv}$.

Theorem 1 *Let X be a graph with n vertices and m edges. Then the F-index and its co-index of* $X_{k}^{++}$ *are calculated as*

(i) $F(X_{k}^{++})={\left(k+1\right)}^{3}F(X)+8km$*.*
(ii) $\bar{F}(X_{k}^{++})=(n+km-1){\left(k+1\right)}^{2}M_{1}(X)-{\left(k+1\right)}^{3}F(X)+4km(n+km-3)$*.*

Proof (i) From the definition of *F*-index, we have

$$F(X_{k}^{++})=\sum_{p\in V(X_{k}^{++})}d^{3}(p/X_{k}^{++})=\sum_{p\in V(X_{k}^{++})\cap V(X)}d^{3}(p/X_{k}^{++})+\sum_{p\in V(X_{k}^{++})\cap E^{\prime }(X)}d^{3}(p/X_{k}^{++})$$

With the help of Table 1

$$=\sum_{p\in V(X)}{\left((k+1)d(p/X)\right)}^{3}+\sum_{e\in E(X)}2^{3}={\left(k+1\right)}^{3}F(X)+8km.$$

**Table 1:** [21] The degree distributions of the point vertices and line vertices in $X_{k}^{uv}$ and $\bar{X_{k}^{uv}}$ are corresponding to that vertices and edges in *X*.

Degrees of point vertices	Degrees of line vertices	Orders	Sizes
$d(p/X_{k}^{++})=(k+1)d(p/X)$	$d(e/X_{k}^{++})=2$	(*km* + *n*)	(2*k* + 1)*m*
$d(p/X_{k}^{+-})=k(m-d(p/X))+d(p/X)$	$d(e/X_{k}^{+-})=(n-2)$	(*km* + *n*)	*m* + (*n* − 2)*km*
$d(p/X_{k}^{-+})=(k-1)d(p/X)+(n-1)$	$d(e/X_{k}^{-+})=2$	(*km* + *n*)	*n*(*n* − 1)/2 − *m*(1 − 2*k*)
$d(p/X_{k}^{--})=(n+km-1)-(k+1)d(p/X)$	$d(e/X_{k}^{--})=(n-2)$	(*km* + *n*)	(*n* − 2)*mk* + *n*(*n* − 1)/2 − *m*
$d(p/\bar{X_{k}^{++}})=n+km-1-(k+1)d(p/X)$	$d(e/\bar{X_{k}^{++}})=n+km-3$	(*km* + *n*)	$\left(\begin{array}{c} n\\ 2 \end{array}\right)-m+km(n-2)+\left(\begin{array}{c} mk\\ 2 \end{array}\right)$
$d(p/\bar{X_{k}^{+-}})=n-1+(k-1)d(p/X)$	$d(e/\bar{X_{k}^{+-}})=(km+1)$	(*km* + *n*)	$\left(\begin{array}{c} n\\ 2 \end{array}\right)-m+2mk+\left(\begin{array}{c} km\\ 2 \end{array}\right)$
$d(p/\bar{X_{k}^{-+}})=km-(k-1)d(p/X)$	$d(e/\bar{X_{k}^{-+}})=n+km-3$	(*km* + *n*)	$m+km(n-2)+\left(\begin{array}{c} km\\ 2 \end{array}\right)$
$d(p/\bar{X_{k}^{--}})=(k+1)d(p/X)$	$d(e/\bar{X_{k}^{--}})=(km+1)$	(*km* + *n*)	$m+2mk+\left(\begin{array}{c} km\\ 2 \end{array}\right)$

(ii) By applying the Proposition 2, Proposition 3, Table 1 and first part of Theorem 1, we get

$\bar{F}(X_{k}^{++})=(n^{⁎}-1)M_{1}(X_{k}^{++})-F(X_{k}^{++})$, where $n^{⁎}$ be the number of vertices of $X_{k}^{++}$

$$=(n+km-1)({\left(k+1\right)}^{2}M_{1}(X)+4km)-({\left(k+1\right)}^{3}F(X)+8km)=(n+km-1){\left(k+1\right)}^{2}M_{1}(X)-{\left(k+1\right)}^{3}F(X)+4km(n+km-3).$$

 □

Corollary 1 *Let X be a graph of order n and size m. Then*

(i) $F(\bar{X_{k}^{++}})=3(n+km-1){\left(k+1\right)}^{2}M_{1}(X)-{\left(k+1\right)}^{3}F(X)+(n+km){\left(n+km-1\right)}^{3}-6m(2k+1){\left(n+km-1\right)}^{2}+12km(n+km-1)-8km$*.*
(ii) $\bar{F}(\bar{X_{k}^{++}})={\left(k+1\right)}^{3}F(X)-2(n+km-1){\left(k+1\right)}^{2}M_{1}(X)+2m(2k+1){\left(n+km-1\right)}^{2}-8km(n+km-2)$*.*

Proof (i) Using the Proposition 2, Proposition 3, Table 1 and Theorem 1, we have

$F(\bar{X_{k}^{++}})=n^{⁎}{\left(n^{⁎}-1\right)}^{3}-6m^{⁎}{\left(n^{⁎}-1\right)}^{2}+3(n^{⁎}-1)M_{1}(X_{k}^{++})-F(X_{k}^{++})$, where $n^{⁎}$ and $m^{⁎}$ be the order and size of $X_{k}^{++}$, respectively.

$$=(n+km){\left(n+km-1\right)}^{3}-6m(2k+1){\left(n+km-1\right)}^{2}\;\;+3(n+km-1)({\left(k+1\right)}^{2}M_{1}(X)+4km)-{\left(k+1\right)}^{3}F(X)-8km=3(n+km-1){\left(k+1\right)}^{2}M_{1}(X)-{\left(k+1\right)}^{3}F(X)+(n+km){\left(n+km-1\right)}^{3}\;\;-6m(2k+1){\left(n+km-1\right)}^{2}+12km(n+km-1)-8km.$$

(ii) Applying the Theorem 1, Table 1 and the Proposition 2, Proposition 3, we have

$$\bar{F}(\bar{X_{k}^{++}})=2m(2k+1){\left(n+km-1\right)}^{2}-(n+km-1)M_{1}(X_{k}^{++})-\bar{F}(X_{k}^{++})=2m(2k+1){\left(n+km-1\right)}^{2}-(n+km-1)({\left(k+1\right)}^{2}M_{1}(X)+4km)-(n+km-1){\left(k+1\right)}^{2}M_{1}(X)+{\left(k+1\right)}^{3}F(X)-4km(n+km-3)={\left(k+1\right)}^{3}F(X)-2(n+km-1){\left(k+1\right)}^{2}M_{1}(X)+2m(2k+1){\left(n+km-1\right)}^{2}-8km(n+km-2).$$

 □

**Figure 2:**
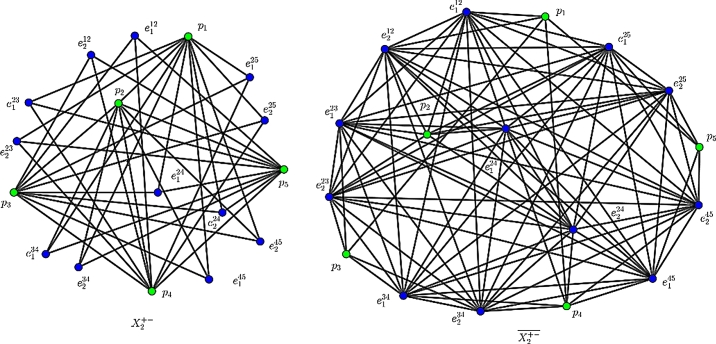
An illustrate of $X_{k}^{+-}$: The 2nd GTG $X_{2}^{+-}$ and its complement $\bar{X_{2}^{+-}}$.

The F-index and its co-index of $X_{k}^{+-}$ (see Fig. 2) are obtained in the following.

Theorem 2 *Let X be a graph of order n and size m. Then*

(i) $F(X_{k}^{+-})=km(3{\left(k-1\right)}^{2}M_{1}(X)-6(k-1)km^{2}+m^{2}k^{2}n+{\left(n-2\right)}^{3})-{\left(k-1\right)}^{3}F(X)$*.*
(ii) $\bar{F}(X_{k}^{+-})={\left(k-1\right)}^{3}F(X)+(n-2km-1){\left(k-1\right)}^{2}M_{1}(X)+6m^{3}k^{2}(k-1)-nm^{3}k^{3}-km{\left(n-2\right)}^{3}+km(n+km-1)(nmk+4m-4km+{\left(n-2\right)}^{2})$*.*

Proof (i) The *F*-index of $X_{k}^{+-}$ can be revealed as

$$F(X_{k}^{+-})=\sum_{p\in V(X_{k}^{+-})}d^{3}(p/X_{k}^{+-})=\sum_{p\in V(X_{k}^{+-})\cap V(X)}d^{3}(p/X_{k}^{+-})+\sum_{p\in V(X_{k}^{+-})\cap E^{\prime }(X)}d^{3}(p/X_{k}^{+-})=\sum_{p\in V(X_{k}^{+-})\cap V(X)}{\left(k(m-d(p/X))+d(p/X)\right)}^{3}+\sum_{p\in V(X_{k}^{+-})\cap E^{\prime }(X)}{\left(n-2\right)}^{3}=\sum_{p\in V(X)}({\left(1-k\right)}^{3}d^{3}(p/X)+3km{\left(k-1\right)}^{2}d^{2}(p/X)+3m^{2}k^{2}(1-k)d(p/X)+m^{3}k^{3})+{\left(n-2\right)}^{3}km={\left(1-k\right)}^{3}F(X)+3km{\left(k-1\right)}^{2}M_{1}(X)-6m^{3}k^{2}(k-1)+m^{3}k^{3}n+{\left(n-2\right)}^{3}mk.$$

(ii) With the help of Proposition 1, Proposition 2, Proposition 3, we can prove it in a similar way. □

Corollary 2 *Consider* X(n,m) *be a graph and* $\bar{X_{k}^{+-}}$ *be the complement of* $X_{k}^{+-}$*. Then*

(i) $F(\bar{X_{k}^{+-}})={\left(k-1\right)}^{3}F(X)+3(n-1){\left(k-1\right)}^{2}M_{1}(X)+(n+km){\left(n+km-1\right)}^{3}-nm^{3}k^{3}-km{\left(n-2\right)}^{3}-6m((n-2)k+1){\left(n+km-1\right)}^{2}+3km(nkm+4m-4km+{\left(n-2\right)}^{2})(n+km-1)+6m^{3}k^{2}(k-1)$*.*
(ii) $\bar{F}(\bar{X_{k}^{+-}})={\left(k-1\right)}^{2}(km-2n+2)M_{1}(X)-{\left(k-1\right)}^{3}F(X)-(n+2km){\left(n-2\right)}^{2}km+2m((n-2)k+1){\left(n+km-1\right)}^{2}-2m^{2}k(kn-4k+4)(n+km-1)+nm^{3}k^{3}-6m^{3}k^{2}(k-1)$*.*

**Figure 3:**
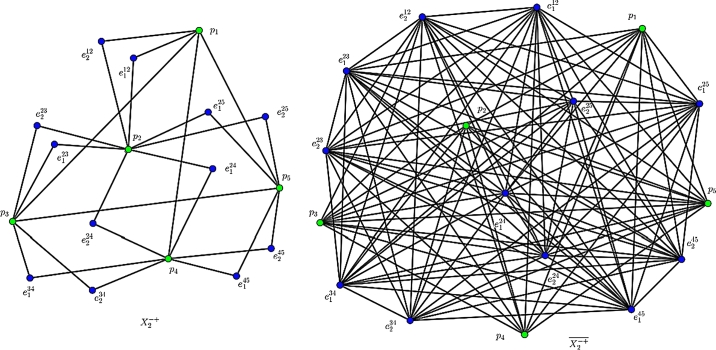
An illustrate of $X_{k}^{-+}$: The 2nd GTG $X_{2}^{-+}$ and its complement $\bar{X_{2}^{-+}}$.

In the following, we obtain the *F*-index of the *k*th GTG $X_{k}^{-+}$ (see Fig. 3).

Theorem 3 *Let X be an* (n,m) *graph. Then F-index and its co-index for* $X_{k}^{-+}$ *are given by*

(i) $F(X_{k}^{-+})={\left(k-1\right)}^{3}F(X)+3{\left(k-1\right)}^{2}(n-1)M_{1}(X)+n{\left(n-1\right)}^{3}+6m{\left(n-1\right)}^{2}(k-1)+8km$*.*
(ii) $\bar{F}(X_{k}^{-+})=(km-2n+2){\left(k-1\right)}^{2}M_{1}(X)-{\left(k-1\right)}^{3}F(X)+2m(n-1)(k-1)(2km-n+1)+km(n^{3}-2n^{2}+4km+5n-12)$*.*

Proof (i) We get from the definition of *F*-index

$$F(X_{k}^{-+})=\sum_{p\in V(X_{k}^{-+})}d^{3}(p/X_{k}^{-+})=\sum_{p\in V(X_{k}^{-+})\cap V(X)}d^{3}(p/X_{k}^{-+})+\sum_{p\in V(X_{k}^{-+})\cap E^{\prime }(X)}d^{3}(p/X_{k}^{-+})=\sum_{p\in V(X_{k}^{-+})\cap V(X)}{\left((k-1)d(p/X)+(n-1)\right)}^{3}+\sum_{p\in V(X_{k}^{-+})\cap E^{\prime }(X)}2^{3}=\sum_{p\in V(X)}((k-1)d^{3}(p/X)+{\left(n-1\right)}^{3}+3{\left(k-1\right)}^{2}(n-1)d^{2}(p/X)+3{\left(n-1\right)}^{2}(k-1)d(p/X))+8km={\left(k-1\right)}^{3}F(X)+n{\left(n-1\right)}^{3}+3{\left(k-1\right)}^{2}(n-1)M_{1}(X)+6m{\left(n-1\right)}^{2}(k-1)+8km.$$

(ii) From Theorem 3, Table 1 and Proposition 1, Proposition 2, Proposition 3 we can get the required expression. □

Corollary 3 *Let us consider X be a graph with n vertices and m edges and* $\bar{X_{k}^{-+}}$ *be the complement of the graph* $X_{k}^{-+}$*. Then*

(i) $F(\bar{X_{k}^{-+}})=3km{\left(k-1\right)}^{2}M_{1}(X)-{\left(k-1\right)}^{3}F(X)+(n+km){\left(n+km-1\right)}^{3}-6m{\left(n-1\right)}^{2}(k-1)-3(n^{2}+4km-n-2m){\left(n+km-1\right)}^{2}+12m(kn-n+1)(n+km-1)+n{\left(n-1\right)}^{2}(2n+3km-2)-8km$*.*
(ii) $\bar{F}(\bar{X_{k}^{-+}})={\left(k-1\right)}^{3}F(X)+{\left(k-1\right)}^{2}(n-2km-1)M_{1}(X)+(n^{2}+4km-n-2m){\left(n+km-1\right)}^{2}-2m(n-1)(k-1)(4k+n-1)-n{\left(n-1\right)}^{2}(2km+n-1)-8km(km+n-2)$*.*

**Figure 4:**
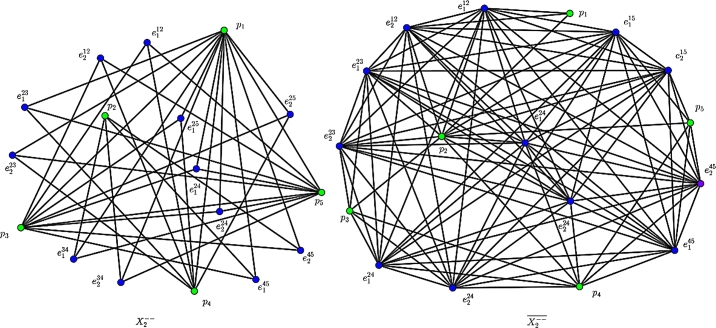
An illustrate of $X_{k}^{--}$: The 2nd GTG $X_{2}^{--}$ and its complement $\bar{X_{2}^{--}}$.

The F-index and its co-index of $X_{k}^{--}$ (see Fig. 4) are obtained in the following.

Theorem 4 *The F-index and its co-index for the kth GTG* $X_{k}^{--}$ *are expressed as*

(i) $F(X_{k}^{--})=n{\left(n+km-1\right)}^{3}-{\left(k+1\right)}^{3}F(X)+3(n+km-1){\left(k+1\right)}^{2}M_{1}(X)+km{\left(n-2\right)}^{3}-6m(k+1)(n+km-1)$*.*
(ii) $\bar{F}(X_{k}^{--})={\left(k+1\right)}^{3}F(X)-2m(k+1)(n+km-1)(2km+2n-5)+km(km+1){\left(n-2\right)}^{2}-2(n+km-1){\left(k+1\right)}^{2}M_{1}(X)$*.*

Proof (i) From the definition of *F*-index, we get

$$F(X_{k}^{--})=\sum_{pq\in E(X_{k}^{--})}(d^{2}(p/X_{k}^{--})+d^{2}(q/X_{k}^{--}))=\sum_{pq\notin E(X)}[{\left\{(n+km-1)-(k+1)d(p/X)\right\}}^{2}+{\left\{(n+km-1)-(k+1)d(q/X)\right\}}^{2}]+\sum_{p\in V(X)}k(m-d(p/X))[\{(n+km-1){-(k+1)d(p/X)\}}^{2}+{\left(n-2\right)}^{2}]=\sum_{pq\notin E(X)}[2{\left(n+km-1\right)}^{2}-2(k+1)(n+km-1)(d(p/X)+d(q/X))+{\left(k+1\right)}^{2}(d^{2}(p/X)+d^{2}(q/X))]+\sum_{p\in V(X)}k(m-d(p/X))[{\left(n+km-1\right)}^{2}+{\left(n-2\right)}^{2}-2(k+1)(n+km-1)d(p/X)+{\left(k+1\right)}^{2}d^{2}(p/X)]=2{\left(n+km-1\right)}^{2}(\left(\begin{array}{c} n\\ 2 \end{array}\right)-m)-2(k+1)(n+km-1)\bar{M_{1}}(X)+{\left(k+1\right)}^{2}\bar{F}(X)+kmn{\left(n+km-1\right)}^{2}+kmn{\left(n-2\right)}^{2}-4m^{2}k(k+1)(n+km-1)+km{\left(k+1\right)}^{2}M_{1}(X)-2km{\left(n+km-1\right)}^{2}-2km{\left(n-2\right)}^{2}+2k(k+1)(n+km-1)M_{1}(X)-k{\left(k+1\right)}^{2}F(X)=n{\left(n+km-1\right)}^{3}-{\left(k+1\right)}^{3}F(X)+3(n+km-1){\left(k+1\right)}^{2}M_{1}(X)-6m(k+1)(n+km-1)+km{\left(n-2\right)}^{3}.$$

(ii) With the help of the Proposition 2, Proposition 3, we can write

$$\bar{F}(X_{k}^{--})=(n+km-1)M_{1}(X_{k}^{--})-F(X_{k}^{--})$$

Using the results of $M_{1}(X_{k}^{--})$ and $F(X_{k}^{--})$

$$=(n+km-1)\{n{\left(n+km-1\right)}^{2}-4m(k+1)(n+km-1)+{\left(k+1\right)}^{2}M_{1}(X)\;+km{\left(n-2\right)}^{2}\}-\{n{\left(n+km-1\right)}^{3}-{\left(k+1\right)}^{3}F(X)\;+3{\left(k+1\right)}^{2}(n+km-1)M_{1}(X)-6m(k+1)(n+km-1)+km{\left(n-2\right)}^{3}\}={\left(k+1\right)}^{3}F(X)-2m(k+1)(n+km-1)(2km+2n-5)\;+km(km+1){\left(n-2\right)}^{2}-2(n+km-1){\left(k+1\right)}^{2}M_{1}(X).$$

 □

Corollary 4 *Let us consider X be a graph with n vertices and m edges and* $\bar{X_{k}^{--}}$ *be the complement of the graph* $X_{k}^{--}$*. Then*

(i) $F(\bar{X_{k}^{--}})={\left(k+1\right)}^{3}F(X)+(3n+km){\left(n+km-1\right)}^{3}-3(n^{2}+2kmn+2m-n){\left(n+km-1\right)}^{2}+6m(k+1)(n+km-1)+km(2n+3km-1){\left(n-2\right)}^{2}$*.*
(ii) $\bar{F}(\bar{X_{k}^{--}})=(n+km-1){\left(k+1\right)}^{2}M_{1}(X)-{\left(k+1\right)}^{3}F(X)-km(n+2km){\left(n-2\right)}^{2}-n{\left(n+km-1\right)}^{3}+(n^{2}+2kmn-n+2m){\left(n+km-1\right)}^{2}+2m(k+1)(2km+2n-5)$*.*

## 4 The Zagreb polynomials on $X_{k}^{++}$

In chemical graph theory, the Zagreb polynomial is one of the degree graph polynomials. In 2009, Fath-Tabar [12] introduced the Zagreb polynomials. The Zagreb polynomials for the graph $X_{k}^{++}$ are computed in the following Theorems and Corollaries.

Theorem 5 *Let X be a graph of order n and size m. Then the Zagreb polynomials of* $X_{k}^{++}$ *are follows as*

(i) $M_{1}(X_{k}^{++},x)=M_{1}(X,x^{\left(k+1\right)})+x^{2}kM_{1}^{⁎}(X,x^{\left(k+1\right)})$*.*
(ii) $M_{2}(X^{++},x)=M_{-\frac{\left(n+km-1\right)}{\left(k+1\right)},-\frac{\left(n+km-1\right)}{\left(k+1\right)}}^{\prime }(X,x^{{\left(k+1\right)}^{2}})+x^{2(n+km-1)}kM_{1}^{⁎}(X,x^{-(k+1)})$*.*
(iii) $M_{3}(X_{k}^{++},x)=M_{3}(X,x^{\left(k+1\right)})+\frac{km}{x^{2}}M_{0}(X,x^{\left(k+1\right)})-\frac{k}{x^{2}}M_{1}^{⁎}(X,x^{(}k+1))+\left(\begin{array}{c} km\\ 2 \end{array}\right)$*.*

Proof (i) The $M_{1}(X_{k}^{++},x)$ polynomial can be expressed as

$$M_{1}(X_{k}^{++},x)=\sum_{pq\in E(X_{k}^{++})}x^{\left(d(p/X_{k}^{++})+d(q/X_{k}^{++})\right)}.$$

The edges of $E(X_{k}^{++})$ can be divided into two parts like $E_{1}^{++/k}=\{pq\in E(X)\}$ and $E_{2}^{++/k}=\{pe|\;\text{the}$ vertex *p* and edge *e* in *X* are incident to each other} such that $|E_{1}^{++/k}|=m$ and $|E_{2}^{++/k}|=2km$, respectively.

$$=\sum_{pq\in E_{1}^{++/k}}x^{\left(k+1)(d(p/X)+d(q/X)\right)}+\sum_{pe\in E_{2}^{++/k}}x^{(k+1)d(p/X)+2}\text{ (Using Table 1)}=M_{1}(X,x^{\left(k+1\right)})+x^{2}k\sum_{p\in V(X)}d(p/X)x^{\left(k+1)d(p/X\right)}=M_{1}(X,x^{\left(k+1\right)})+x^{2}kM_{1}^{⁎}(X,x^{\left(k+1\right)}).$$

(ii) Now,

$$M_{2}(X_{k}^{++},x)=\sum_{pq\in E(X_{k}^{++})}x^{d(p/X_{k}^{++})d(q/X_{k}^{++})}=\sum_{pq\in E(X)}x^{\left((n+km-1)-(k+1)d(p/X))((n+km-1)+d(q/X)\right)}+\sum_{pe\in E_{2}^{++/k}}x^{2((n+km-1)-(k+1)d(p/X))}=M_{-\frac{\left(n+km-1\right)}{\left(k+1\right)},-\frac{\left(n+km-1\right)}{\left(k+1\right)}}^{\prime }(X,x^{{\left(k+1\right)}^{2}})+x^{2(n+km-1)}k\sum_{p\in V(X)}d(p/X)x^{-(k+1)d(p/X)}=M_{-\frac{\left(n+km-1\right)}{\left(k+1\right)},-\frac{\left(n+km-1\right)}{\left(k+1\right)}}^{\prime }(X,x^{{\left(k+1\right)}^{2}})+x^{2(n+km-1)}kM_{1}^{⁎}(X,x^{-(k+1)}).$$

(iii) From the definition of $M_{3}(X,x)$ and putting $X=X_{k}^{++}$, we have

$$M_{3}(X_{k}^{++},x)=\sum_{pq\in E(X_{k}^{++})}x^{\left|d(p/X_{k}^{++})-d(q/X_{k}^{++})\right|}=\sum_{pq\in E(X)}x^{\left|(k+1)d(p/X)-(k+1)d(q/X)\right|}+\sum_{pe\in E_{2}^{++/k}}x^{|(k+1)d(p/X)|-2}+\sum_{ef\in E_{3}^{++/k}}x^{0}=M_{3}(X,x^{\left(k+1\right)})+\frac{k}{x^{2}}\sum_{p\in V(X)}(m-d(p/x))x^{\left(k+1)d(p/X\right)}+\left(\begin{array}{c} km\\ 2 \end{array}\right)=M_{3}(X,x^{\left(k+1\right)})+\frac{km}{x^{2}}M_{0}(X,x^{(}k+1))-\frac{k}{x^{2}}M_{1}^{⁎}(X,x^{\left(k+1\right)})+\left(\begin{array}{c} km\\ 2 \end{array}\right).$$

 □

Corollary 5 *The Zagreb polynomials for the graph* $\bar{X_{k}^{++}}$ *are expressed as*

(i) $M_{1}(\bar{X_{k}^{++}},x)=x^{2(n+km-1)}\bar{M_{1}}(X,x^{-(k+1)})+kmx^{2(n+km-2)}M_{0}(X,x^{-(k+1)})-kx^{2(n+km-2)}M_{1}^{⁎}(X,x^{-(k+1)})+\left(\begin{array}{c} km\\ 2 \end{array}\right)x^{2(n+km-3)}$*.*
(ii) $M_{2}(\bar{X_{k}^{++}},x)={\bar{M^{\prime }}}_{-\frac{\left(n+km-1\right)}{\left(k+1\right)},-\frac{\left(n+km-1\right)}{\left(k+1\right)}}(X,x^{{\left(k+1\right)}^{2}})+kmx^{\left(n+km-1)(n+km-3\right)}\times M_{0}(X,x^{-(k+1)(n+km-3)})-tx^{\left(n+km-1)(n+km-3\right)}M_{1}^{⁎}(X,x^{-(k+1)(n+km-3)})+\left(\begin{array}{c} km\\ 2 \end{array}\right)x^{{\left(n+km-3\right)}^{2}}$*.*
(iii) $M_{3}(\bar{X_{k}^{++}},x)=\bar{M_{3}}(X,x^{\left(k+1\right)})+\frac{km}{x^{2}}M_{0}(X,x^{\left(k+1\right)})-\frac{k}{x^{2}}M_{1}^{⁎}(X,x^{\left(k+1\right)})+\left(\begin{array}{c} km\\ 2 \end{array}\right)$*.*

Proof (i) The edges of $E(\bar{X_{k}^{++}})$ can be partitioned into three parts such as $\bar{E_{1}^{++/k}}=\{pq\notin E(X)\}$, $\bar{E_{2}^{++/k}}=\{pe|\;\text{the}$ vertex *p* and edge *e* in *X* are not incident to each other} and $\bar{E_{3}^{++/k}}=\{ef|e,f\in E(X)\}$ such that $|\bar{E_{1}^{++/k}}|=\left(\begin{array}{c} n\\ 2 \end{array}\right)-m$, $\left|\bar{E_{2}^{++/k}}|=km(n-2\right)$ and $|\bar{E_{3}^{++/k}}|=\left(\begin{array}{c} km\\ 2 \end{array}\right)$, respectively.

By the definition of the polynomial $M_{1}(X,x)$ and setting $X=\bar{X_{k}^{++}}$, we get

$$M_{1}(\bar{X_{k}^{++}},x)=\sum_{pq\in E(\bar{X_{k}^{++}})}x^{d(p/\bar{X_{k}^{++}})+d(q/\bar{X_{k}^{++}})}=\sum_{pq\in \bar{E_{1}^{++/k}}}x^{d(p/\bar{X_{k}^{++}})+d(q/\bar{X_{k}^{++}})}+\sum_{pe\in \bar{E_{2}^{++/k}}}x^{d(p/\bar{X_{k}^{++}})+d(q/\bar{X_{k}^{++}})}+\sum_{ef\in \bar{E_{3}^{++/k}}}x^{d(p/\bar{X_{k}^{++}})+d(q/\bar{X_{k}^{++}})}=\sum_{pq\notin E(X)}x^{\left[2(n+km-1)-(k+1)(d(p/X)+d(q/X))\right]}+\sum_{pe\in \bar{E_{2}^{++/k}}}x^{2(n+km-2)-(k+1)d(p/X)}+\sum_{ef\in \bar{E_{3}^{++/k}}}x^{2(n+km-3)}=x^{2(n+km-1)}\sum_{pq\notin E(X)}x^{-(k+1)(d(p/X)+d(q/X))}+k\sum_{p\in V(X)}(m-d(p/X))x^{2(n+km-2)-(k+1)d(p/X)}+\left(\begin{array}{c} km\\ 2 \end{array}\right)x^{2(n+km-3)}=x^{2(n+km-1)}\bar{M_{1}}(X,x^{-(k+1)})+kmx^{2(n+km-2)}M_{0}(X,x^{-(k+1)})-kx^{2(n+km-2)}M_{1}^{⁎}(X,x^{-(k+1)})+\left(\begin{array}{c} km\\ 2 \end{array}\right)x^{2(n+km-3)}.$$

(ii) From definition, we have

$$M_{2}(\bar{X_{k}^{++}},x)=\sum_{pq\in E(\bar{X_{k}^{++}})}x^{d(p/\bar{X_{k}^{++}})d(q/\bar{X_{k}^{++}})}=\sum_{pq\in \bar{E_{1}^{++/k}}}x^{d(p/\bar{X_{k}^{++}})d(q/\bar{X_{k}^{++}})}+\sum_{pe\in \bar{E_{2}^{++/k}}}x^{d(p/\bar{X_{k}^{++}})d(e/\bar{X_{k}^{++}})}+\sum_{ef\in \bar{E_{3}^{++/k}})}x^{d(e/\bar{X_{k}^{++}})d(f/\bar{X_{k}^{++}})}=\sum_{pq\notin E(X)}x^{\left(n+km-1-(k+1)d(p/X))(n+km-1-(k+1)d(q/X)\right)}+\sum_{ef\in E(\bar{E_{3}^{++/k}})}x^{{\left(n+km-3\right)}^{2}}+x^{\left(n+km-1)(n+km-3\right)}k\sum_{p\in V(X)}(m-d(p/X))x^{-(k+1)(n+km-3)d(p/X)}={\bar{M^{\prime }}}_{-\frac{\left(n+km-1\right)}{\left(k+1\right)},-\frac{\left(n+km-1\right)}{\left(k+1\right)}}(X,x^{{\left(k+1\right)}^{2}})+kmx^{\left(n+km-1)(n+km-3\right)}M_{0}(X,x^{-(k+1)(n+km-3)})+\left(\begin{array}{c} km\\ 2 \end{array}\right)x^{{\left(n+km-3\right)}^{2}}-kx^{\left(n+km-1)(n+km-3\right)}M_{1}^{⁎}(X,x^{-(k+1)(n+km-3)}).$$

(iii) Now,

$$M_{3}(\bar{X_{k}^{++}},x)=\sum_{pq\in E(\bar{X_{k}^{++}})}x^{\left|d(p/\bar{X_{k}^{++}})-d(q/\bar{X_{k}^{++}})\right|}=\sum_{pq\in E(\bar{X_{1}^{++/k}})}x^{\left|d(p/\bar{X_{k}^{++}})-d(q/\bar{X_{k}^{++}})\right|}+\sum_{pe\in E(\bar{X_{2}^{++/k}})}x^{\left|d(p/\bar{X_{k}^{++}})-d(e/\bar{X_{k}^{++}})\right|}+\sum_{ef\in E(\bar{X_{3}^{++/k}})}x^{\left|d(e/\bar{X_{k}^{++}})-d(f/\bar{X_{k}^{++}})\right|}=\sum_{pq\notin E(X)}x^{\left(k+1)|d(p/X)-d(q/X)\right|}+k\sum_{p\in V(X)}(m-d(p/X))x^{\left|(n+km-1)-(k+1)d(p/X)-(n+km-3)\right|}+\sum_{ef\in E(\bar{X_{3}^{++}})}x^{0}=\bar{M_{3}}(X,x^{\left(k+1\right)})+\frac{km}{x^{2}}M_{0}(X,x^{\left(k+1\right)})-\frac{k}{x^{2}}M_{1}^{⁎}(X,x^{\left(k+1\right)})+\left(\begin{array}{c} km\\ 2 \end{array}\right).$$

 □

Corollary 6

(i) $\bar{M_{1}}(X_{k}^{++},x)=\bar{M_{1}}(X,x^{\left(k+1\right)})+kmx^{2}M_{0}(X,x^{\left(k+1\right)})-x^{2}kM_{1}^{⁎}(X,x^{\left(k+1\right)})+\left(\begin{array}{c} km\\ 2 \end{array}\right)x^{4}$*.*
(ii) $\bar{M_{2}}(X_{k}^{++},x)=\bar{M_{2}}(X,x^{{\left(k+1\right)}^{2}})+mkM_{0}(X,x^{2(k+1)})-kM_{1}^{⁎}(X,x^{2(k+1)})+\left(\begin{array}{c} km\\ 2 \end{array}\right)x^{4}$*.*
(iii) $\bar{M_{3}}(X_{k}^{++},x)=\bar{M_{3}}(X,x^{\left(k+1\right)})+\frac{km}{x^{2}}M_{0}(X,x^{\left(k+1\right)})-\frac{k}{x^{2}}M_{1}^{⁎}(X,x^{\left(k+1\right)})+\left(\begin{array}{c} km\\ 2 \end{array}\right)$*.*

Proof (i)

$$\bar{M_{1}}(X_{k}^{++},x)=\sum_{pq\notin E(X_{k}^{++})}x^{\left(d(p/X_{k}^{++})+d(q/X_{k}^{++})\right)}=\sum_{pq\in \bar{E_{1}^{++/k}}}x^{\left(k+1)(d(p/X)+d(q/X)\right)}+\sum_{pe\in E_{2}^{\bar{++/k}}}x^{(k+1)d(p/X)+2}+\sum_{ef\in E_{3}^{\bar{++/k}}}x^{4}$$

The partition of the edge set $\bar{E(X_{k}^{++})}$ follows as Corollary 5

$$=\sum_{pq\notin E(X)}x^{\left(k+1)(d(p/X)+d(q/X)\right)}\;\;+x^{2}k\sum_{p\in V(X)}(m-d(p/X))x^{\left(k+1)d(p/X\right)}+\left(\begin{array}{c} mk\\ 2 \end{array}\right)x^{4}=\bar{M_{1}}(X,x^{\left(k+1\right)})+x^{2}k[mM_{0}(X,x^{\left(k+1\right)})-M_{1}^{⁎}(X,x^{\left(k+1\right)}]+\left(\begin{array}{c} mk\\ 2 \end{array}\right)x^{4}.$$

(ii)

$$\bar{M_{2}}(X_{k}^{++},x)=\sum_{pq\notin E(X_{k}^{++})}x^{d(p/X_{k}^{++})d(q/X_{k}^{++})}=\sum_{pq\in (\bar{E_{1}^{++/k}})}x^{d(p/X_{k}^{++})d(q/X_{k}^{++})}+\sum_{pe\in (\bar{E_{2}^{++/k}})}x^{d(p/X_{k}^{++})d(e/X_{k}^{++})}+\sum_{ef\in (\bar{E_{3}^{++/k}})}x^{d(e/X_{k}^{++})d(f/X_{k}^{++})}=\sum_{pq\notin E(X)}x^{{\left(k+1\right)}^{2}d(p/X)d(q/X)}+k\sum_{p\in V(X)}(m-d(p/X))x^{2(k+1)d(p/X)}+\sum_{ef\in (E_{3}^{++/k})}x^{4}=\bar{M_{2}}(X,x^{{\left(k+1\right)}^{2}})+k(mM_{0}(X,x^{2(k+1)})-M_{1}^{⁎}(X,x^{2(k+1)}))+\left(\begin{array}{c} km\\ 2 \end{array}\right)x^{4}.$$

(iii)

$$\bar{M_{3}}(X_{k}^{++},x)=\sum_{pq\notin E(X_{k}^{++})}x^{\left|d(p/X_{k}^{++})-d(q/X_{k}^{++})\right|}=\sum_{pq\in (\bar{E_{1}^{++/k}})}x^{\left|d(p/X_{k}^{++})-d(q/X_{k}^{++})\right|}+\sum_{pe\in (\bar{E_{2}^{++/k}})}x^{\left|d(p/X_{k}^{++})-d(e/X_{k}^{++})\right|}+\sum_{ef\in (\bar{E_{3}^{++/k}})}x^{\left|d(e/X_{k}^{++})-d(f/X_{k}^{++})\right|}=\sum_{pq\notin E(X)}x^{\left|(k+1)d(p/X)-(k+1)d(q/X)\right|}+\frac{k}{x^{2}}\sum_{p\in V(X)}(m-d(p/X))x^{\left(k+1)d(p/X\right)}+\left(\begin{array}{c} km\\ 2 \end{array}\right)=\bar{M_{3}}(X,x^{\left(k+1\right)})+\frac{km}{x^{2}}M_{0}(X,x^{\left(k+1\right)})-\frac{k}{x^{2}}M_{1}^{⁎}(X,x^{\left(k+1\right)})+\left(\begin{array}{c} km\\ 2 \end{array}\right).$$

 □

Corollary 7

(i) $\bar{M_{1}}(\bar{X_{k}^{++}},x)=x^{2(n+km-1)}M_{1}(X,x^{-(k+1)})+kx^{2(n+km-2)}M_{1}^{⁎}(X,x^{-(k+1)})$*.*
(ii) $\bar{M_{2}}(\bar{X_{k}^{++}},x)=M_{-\frac{\left(n+km-1\right)}{\left(k+1\right)},-\frac{\left(n+km-1\right)}{\left(k+1\right)}}^{\prime }(X,x^{{\left(k+1\right)}^{2}})+kx^{\left(n+km-1)(n+km-3\right)}\times M_{1}^{⁎}(X,x^{-(k+1)(n+km-3)})$*.*
(iii) $\bar{M_{3}}(\bar{X_{k}^{++}},x)=M_{3}(X,x^{\left(k+1\right)})+\frac{k}{x^{2}}M_{1}^{⁎}(X,x^{\left(k+1\right)})$*.*

Proof (i) With the help of the definition $\bar{M_{1}}(X,x)$ and putting $X=\bar{X_{k}^{++}}$, we get

$$\bar{M_{1}}(\bar{X_{k}^{++}},x)=\sum_{pq\notin E(\bar{X_{k}^{++}})}x^{\left(d(p/\bar{X_{k}^{++}})+d(q/\bar{X_{k}^{++}})\right)}=\sum_{pq\in E(X)}x^{\left(n+km-1-(k+1)d(p/X)+n+km-1-(k+1)d(q/x)\right)}+k\sum_{p\in V(X)}d(p/X)x^{\left(n+km-1(k+1)d(p/X)+n+km-3\right)}=x^{2(n+km-1)}M_{1}(X,x^{-(k+1)})+kx^{2(n+km-2)}M_{1}^{⁎}(X,x^{-(k+1)}).$$

(ii)

$$\bar{M_{2}}(\bar{X_{k}^{++}},x)=\sum_{pq\notin E(\bar{X_{k}^{++}})}x^{\left(d(p/\bar{X_{k}^{++}})d(q/\bar{X_{k}^{++}})\right)}=\sum_{pq\in E(X)}x^{\left(n+km-1-(k+1)d(p/X))(n+km-1-(k+1)d(q/x)\right)}+k\sum_{p\in V(X)}d(p/X)x^{\left(n+km-1-(k+1)d(p/X))(n+km-3\right)}=\sum_{pq\in E(X)}x^{{\left(k+1\right)}^{2}(d(p/X)-\frac{\left(n+km-1\right)}{\left(k+1\right)})(d(q/X)-\frac{\left(n+km-1\right)}{\left(k+1\right)})}+kx^{\left(n+km-1)(n+km-3\right)}\sum_{p\in V(X)}d(p/X)x^{-(n+km-1)(k+1)d(p/X)}=M_{-\frac{\left(n+km-1\right)}{\left(k+1\right)},-\frac{\left(n+km-1\right)}{\left(k+1\right)}}^{\prime }(X,x^{{\left(k+1\right)}^{2}})+kx^{\left(n+km-1)(n+km-3\right)}M_{1}^{⁎}(X,x^{-(k+1)(n+km-3)}).$$

(iii)

$$\bar{M_{3}}(\bar{X_{k}^{++}},x)=\sum_{pq\notin E(\bar{X_{k}^{++}})}x^{\left|d(p/\bar{X_{k}^{++}})-d(q/\bar{X_{k}^{++}})\right|}=\sum_{pq\in E(X)}x^{\left|(k+1)d(p/X)-(k+1)d(q/X)\right|}+\frac{k}{x^{2}}\sum_{p\in V(X)}d(p/X)x^{\left(k+1)d(p/X\right)}=M_{3}(X,x^{\left(k+1\right)})+\frac{k}{x^{2}}M_{1}^{⁎}(X,x^{\left(k+1\right)}).$$

 □

## 5 The Zagreb polynomials on $X_{k}^{+-}$

The expressions for the Zagreb polynomials of the *k*th GTG $X_{k}^{+-}$ are obtained as follows here.

Theorem 6 *For the kth GTG* $X_{k}^{+-}$*, the Zagreb polynomials are*

(i) $M_{1}(X_{k}^{+-},x)=x^{2km}M_{1}(X,x^{-(k-1)})+kmx^{\left(n+km-2\right)}M_{0}(X,x^{-(k-1)})-kx^{\left(n+km-2\right)}M_{1}^{⁎}(X,x^{-(k-1)})$*.*
(ii) $M_{2}(X_{k}^{+-},x)=M_{-\frac{km}{\left(k-1\right)},-\frac{km}{\left(k-1\right)}}^{\prime }(X,x^{\left(k-1\right)})+kmx^{(n-2)km}M_{0}(X,x^{-(n-2)(k-1)})-kx^{(n-2)km}M_{1}^{⁎}(X,x^{-(n-2)(k-1)})$*.*
(iii) $M_{3}(X_{k}^{+-},x)=M_{3}(X,x^{\left(k-1\right)})+kmx^{\left|n-km-2\right|}M_{0}(X,x^{\left(k-1\right)})-kx^{\left|n-km-2\right|}M_{1}^{⁎}(X,x^{\left(k-1\right)})$*.*

Proof (i) The edges of $E(X_{k}^{+-})$ can be separated into two parts like $E_{1}^{+-/k}=\{pq\in E(X)\}$ and $E_{2}^{+-/k}=\{pe|\;\text{the}$ vertex *p* and edge *e* are not incident to each other inX} such that $|E_{1}^{+-/k}|=m$ and $\left|E_{2}^{+-/k}|=km(n-2\right)$, respectively.

By the definition of $M_{1}(X,x)$ and putting $X=X_{k}^{+-}$, we have

$$M_{1}(X_{k}^{+-},x)=\sum_{pq\in E(X_{k}^{+-})}x^{\left(d(p/X_{k}^{+-})+d(q/X_{k}^{+-})\right)}=\sum_{pq\in E_{1}^{+-/k}}x^{2km-(k-1)(d(p/X)+d(q/X))}+\sum_{pe\in E_{2}^{+-/k}}x^{km+n-2-(k-1)d(p/X)}\;(\text{Using Table 1})=x^{2km}\sum_{pq\in E(X)}x^{-(k-1)(d(p/X)+d(q/X))}+kx^{\left(km+n-2\right)}\sum_{p\in V(X)}(m-d(p/X))x^{-(k-1)d(p/X)}=x^{2km}M_{1}(X,x^{-(k-1)})+kmx^{\left(n+km-2\right)}M_{0}(X,x^{-(k-1)})-kx^{\left(n+km-2\right)}M_{1}^{⁎}(X,x^{-(k-1)}).$$

The rest part of the theorem can be established in a similar way. □

Corollary 8

(i) $M_{1}(\bar{X_{k}^{+-}},x)=x^{2(n-1)}\bar{M_{1}}(X,x^{\left(k-1\right)})+kx^{\left(n+km\right)}M_{1}^{⁎}(X,x^{\left(k-1\right)})+\left(\begin{array}{c} mk\\ 2 \end{array}\right)x^{2(km+1)}$*.*
(ii) $M_{2}(\bar{X_{k}^{+-}},x)={\bar{M^{\prime }}}_{\frac{\left(n-1\right)}{\left(k-1\right)},\frac{\left(n-1\right)}{\left(k-1\right)}}(X,x^{{\left(k-1\right)}^{2}})+kx^{\left(n-1)(km+1\right)}M_{1}^{⁎}(X,x^{\left(k-1)(km+1\right)})\;+\left(\begin{array}{c} km\\ 2 \end{array}\right)x^{{\left(km+1\right)}^{2}}$*.*
(iii) $M_{3}(\bar{X_{k}^{+-}},x)=\bar{M_{3}}(X,x^{\left(k-1\right)})+kx^{\left|n-km-2\right|}M_{1}^{⁎}(X,x^{\left(k-1\right)})+\left(\begin{array}{c} km\\ 2 \end{array}\right)$*.*

Proof (i) The edges of $E(\bar{X_{k}^{+-}})$ can be divided into three parts such as $\bar{E_{1}^{+-/k}}=\{pq\notin E(X)\}$, $\bar{E_{2}^{+-/k}}=\{pe|\;\text{the}$ vertex *p* is incident to the edge *e* in *X* } and $\bar{E_{3}^{+-/k}}=\{ef|e,f\in E(X)\}$ such that $|\bar{E_{1}^{+-/k}}|=\left(\begin{array}{c} n\\ 2 \end{array}\right)-m$, $|\bar{E_{2}^{+-/k}}|=2km$ and $|\bar{E_{3}^{+-/k}}|=\left(\begin{array}{c} km\\ 2 \end{array}\right)$, respectively.

By the definition of the polynomial $M_{1}(X,x)$ and setting $X=\bar{X_{k}^{+-}}$, we get

$$M_{1}(\bar{X_{k}^{+-}},x)=\sum_{pq\in E(\bar{X_{k}^{+-}})}x^{d(p/\bar{X_{k}^{+-}})+d(q/\bar{X_{k}^{+-}})}=\sum_{pq\in \bar{E_{1}^{+-/k}}}x^{d(p/\bar{X_{k}^{+-}})+d(q/\bar{X_{k}^{+-}})}+\sum_{pe\in \bar{E_{2}^{+-/k}}}x^{d(p/\bar{X_{k}^{+-}})+d(q/\bar{X_{k}^{+-}})}+\sum_{ef\in \bar{E_{3}^{+-/k}}}x^{d(p/\bar{X_{t}^{+-}k})+d(q/\bar{X_{k}^{+-}})}=\sum_{pq\notin E(X)}x^{\left[2(n-1)+(k-1)(d(p/X)+d(q/X))\right]}+\sum_{pe\in \bar{E_{2}^{+-/k}}}x^{\left(n+km)+(k-1)d(p/X\right)}+\sum_{ef\in \bar{E_{3}^{+-/k}}}x^{2(km+1)}=x^{2(n-1)}\sum_{pq\notin E(X)}x^{\left(k-1)(d(p/X)+d(q/X)\right)}+k\sum_{p\in V(X)}d(p/X)x^{\left(n+km)+(k-1)d(p/X\right)}+\sum_{ef\in \bar{E_{3}^{+-/k}}}x^{2(km+1)}=x^{2(n-1)}\bar{M_{1}}(X,x^{\left(k-1\right)})+kx^{\left(n+km\right)}M_{1}^{⁎}(X,x^{\left(k-1\right)})+\left(\begin{array}{c} km\\ 2 \end{array}\right)x^{2(km+1)}.$$

Similarly, we can prove the rest Corollary 8. □

Corollary 9

(i) ${\bar{M}}_{1}(X_{k}^{+-},x)=x^{2km}\bar{M_{1}}(X,x^{-(k-1)})+kx^{\left(n+km-2\right)}M_{1}^{⁎}(X,x^{-(k-1)})+\left(\begin{array}{c} mk\\ 2 \end{array}\right)x^{2(n-2)}$*.*
(ii) ${\bar{M}}_{2}(X_{k}^{+-},x)={\bar{M^{\prime }}}_{-\frac{km}{\left(k-1\right)},-\frac{km}{\left(k-1\right)}}(X,x^{{\left(k-1\right)}^{2}})+kx^{(n-2)km}M_{1}^{⁎}(X,x^{-(n-2)(k-1)})+\left(\begin{array}{c} km\\ 2 \end{array}\right)x^{{\left(n-2\right)}^{2}}$*.*
(iii) ${\bar{M}}_{3}(X_{k}^{+-},x)=\bar{M_{3}}(X,x^{\left(k-1\right)})+kx^{\left|n-km-2\right|}M_{1}^{⁎}(X,x^{\left(k-1\right)})+\left(\begin{array}{c} km\\ 2 \end{array}\right)$*.*

Corollary 10

(i) ${\bar{M}}_{1}(\bar{X_{k}^{+-}},x)=x^{2(n-1)}M_{1}(X,x^{\left(k-1\right)})+kmx^{\left(n+km\right)}M_{0}(X,x^{\left(k-1\right)})-kx^{\left(n+km\right)}M_{1}^{⁎}(X,x^{\left(k-1\right)})$*.*
(ii) ${\bar{M}}_{2}(\bar{X_{k}^{+-}},x)=M_{\frac{\left(n-1\right)}{\left(k-1\right)},\frac{\left(n-1\right)}{\left(k-1\right)}}^{\prime }(X,x^{{\left(k-1\right)}^{2}})+kmx^{\left(n-1)(km+1\right)}M_{0}(X,x^{\left(k-1))(km+1\right)})-kx^{\left(n-1)(km+1\right)}M_{1}^{⁎}(X,x^{\left(k-1)(km+1\right)})$*.*
(iii) ${\bar{M}}_{3}(\bar{X_{k}^{+-}},x)=M_{3}(X,x^{\left(k-1\right)})+kx^{\left|n-km-2\right|}M_{1}^{⁎}(X,x^{\left(k-1\right)})$*.*

## 6 The Zagreb polynomials on $X_{k}^{-+}$

The $X_{k}^{-+}$ is a one kind of the *k*th GTG of *X*. The F-index and its co-index and also the Zagreb polynomials for the $X_{k}^{-+}$ and its complements are obtained in the following theorems.

The two parts of the edges $E(X_{k}^{-+})$ are $E_{1}^{-+/k}=\{pq\notin E(X)\}$ and $E_{2}^{-+/k}=\{pe|\;\text{the}$ vertex *p* and edge *e* in *X* are incident to each other} such that $|E_{1}^{-+/k}|=\left(\begin{array}{c} n\\ 2 \end{array}\right)-m$ and $|E_{2}^{-+/k}|=2km$, respectively. Analogously,

$$\bar{E(X_{k}^{-+})}=\bar{E_{1}^{-+/k}}\cup \bar{E_{2}^{-+/k}}\cup \bar{E_{3}^{-+/k}}.$$

Also, $|\bar{E_{1}^{-+/k}}|=m$, $\left|\bar{E_{2}^{-+/k}}|=km(n-2\right)$ and $|E_{3}^{-+/k}|=\left(\begin{array}{c} km\\ 2 \end{array}\right)$, respectively.

Theorem 7 *The Zagreb polynomials of* $X_{k}^{-+}$ *are given by*

(i) $M_{1}(X_{k}^{-+},x)=x^{2(n-1)}\bar{M_{1}}(X,x^{\left(k-1\right)})+kx^{\left(n+1\right)}M_{1}^{⁎}(X,x^{\left(k-1\right)})$*.*
(ii) $M_{2}(X_{k}^{-+},x)={\bar{M^{\prime }}}_{\frac{\left(n-1\right)}{\left(k-1\right)},\frac{\left(n-1\right)}{\left(k-1\right)}}(X,x^{{\left(k-1\right)}^{2}})+kx^{2(n-1)}M_{1}^{⁎}(X,x^{2(k-1)})$*.*
(iii) $M_{3}(X_{k}^{-+},x)=\bar{M_{3}}(X,x^{\left(k-1\right)})+kx^{\left|n-3\right|}M_{1}^{⁎}(X,x^{\left(k-1\right)})$*.*

Corollary 11

(i) $M_{1}(\bar{X_{k}^{-+}})=x^{2km}M_{1}(X,x^{-(k-1)})+kmx^{\left(n+2km-3\right)}M_{0}(X,x^{-(k-1)})+\left(\begin{array}{c} km\\ 2 \end{array}\right)x^{2(n+km-3)}-kx^{\left(n+2km-3\right)}M_{1}^{⁎}(X,x^{-(k-1)})$*.*
(ii) $M_{2}(\bar{X_{k}^{-+}})=M_{-\frac{km}{\left(k-1\right)},-\frac{km}{\left(k-1\right)}}^{\prime }(X,x^{{\left(k-1\right)}^{2}})+kmx^{km(n+km-3)}M_{0}(X,x^{-(k-1)(n+km-3)})+\left(\begin{array}{c} km\\ 2 \end{array}\right)x^{{\left(n+km-3\right)}^{2}}-kx^{km(n+km-3)}M_{1}^{⁎}(X,x^{-(k-1)(n+km-3)})$*.*
(iii) $M_{3}(\bar{X_{k}^{-+}})=M_{3}(X,x^{\left(k-1\right)})+kmx^{\left|n-3\right|}M_{0}(X,x^{\left(k-1\right)})-kx^{\left|n-3\right|}M_{1}^{⁎}(X,x^{\left(k-1\right)})+\left(\begin{array}{c} km\\ 2 \end{array}\right)$*.*

Corollary 12

(i) ${\bar{M}}_{1}(X_{k}^{-+},x)=x^{2(n-1)}M_{1}(X,x^{\left(k-1\right)})+kmx^{\left(n+1\right)}M_{0}(X,x^{\left(k-1\right)})-kx^{\left(n+1\right)}M_{1}^{⁎}(X,x^{\left(k-1\right)})+\left(\begin{array}{c} km\\ 2 \end{array}\right)x^{4}$*.*
(ii) ${\bar{M}}_{2}(X_{k}^{-+},x)=M_{\frac{\left(n-1\right)}{\left(k-1\right)},\frac{\left(n-1\right)}{\left(k-1\right)}}^{\prime }(X,x^{{\left(k-1\right)}^{2}})+kmx^{2(n-1)}M_{0}(X,x^{2(k-1)})-kx^{2(n-1)}M_{1}^{⁎}(X,x^{2(k-1)})+\left(\begin{array}{c} km\\ 2 \end{array}\right)x^{4}$*.*
(iii) ${\bar{M}}_{3}(X_{k}^{-+},x)=M_{3}(X,x^{\left(k-1\right)})+kmx^{\left|n-3\right|}M_{0}(X,x^{\left(k-1\right)})-kx^{\left|n-3\right|}\times M_{1}^{⁎}(X,x^{\left(k-1\right)})+\left(\begin{array}{c} km\\ 2 \end{array}\right)$*.*

Corollary 13

(i) ${\bar{M}}_{1}(\bar{X_{k}^{-+}},x)=x^{2km}\bar{M_{1}}(X,x^{-(k-1)})+kx^{\left(n+2km-3\right)}M_{1}^{⁎}(X,x^{-(k-1)})$*.*
(ii) ${\bar{M}}_{2}(\bar{X_{k}^{-+}},x)={\bar{M^{\prime }}}_{-\frac{km}{\left(k-1\right)},-\frac{km}{\left(k-1\right)}}(X,x^{{\left(k-1\right)}^{2}})+kx^{km(n+km-3)}M_{1}^{⁎}(X,x^{-(k-1)(n+km-3)})$*.*
(iii) ${\bar{M}}_{3}(\bar{X_{k}^{-+}},x)=\bar{M_{3}}(X,x^{\left(k-1\right)})+kx^{\left|n-3\right|}M_{1}^{⁎}(X,x^{\left(k-1\right)})$*.*

## 7 The Zagreb polynomials on $X_{k}^{--}$

There exist four *k*th GTG of a graph for the 2-permutation of {+,−}. The $X_{k}^{--}$ is one kind of that four graphical transformations of the graph *X*.

The two subset of the edge set $E(X_{k}^{--})$ are $E_{1}^{--/k}=\{pq\notin E(X)\}$ and $E_{2}^{--/k}=\{pe|\;\text{the}$ vertex *p* is not incident to the edge *e* in *X*} where $|E_{1}^{--/k}|=\left(\begin{array}{c} n\\ 2 \end{array}\right)-m$ and $\left|E_{2}^{--/k}|=km(n-2\right)$, respectively. Similarly,

$$\bar{E(X_{k}^{--})}=\{\bar{E_{1}^{--/k}}=pq\in E(X)\}\cup \{\bar{E_{2}^{--/k}}=pe|\;\text{the vertex }p\text{ is incident to the edge }e\text{ in }X\}\cup \{\bar{E_{3}^{--/k}}=ef|e,f\in E(X)\}.$$

Also, $|\bar{E_{1}^{--/k}}|=m$, $|\bar{E_{2}^{--/k}}|=2km$ and $|E_{3}^{--/k}|=\left(\begin{array}{c} km\\ 2 \end{array}\right)$, respectively.

Theorem 8

(i) $M_{1}(X_{k}^{--},x)=x^{2(n+km-1)}\bar{M_{1}}(X,x^{-(k+1)})+kmx^{\left(2n+km-3\right)}M_{0}(X,x^{-(k+1)})-kx^{\left(2n+km-3\right)}M_{1}^{⁎}(X,x^{-(k+1)})$*.*
(ii) $M_{2}(X_{k}^{--},x)={\bar{M^{\prime }}}_{-\frac{\left(n+km-1\right)}{\left(k+1\right)},-\frac{\left(n+km-1\right)}{\left(k+1\right)}}(X,x^{{\left(k+1\right)}^{2}})+kmx^{\left(n-2)(n+km-1\right)}\times M_{0}(X,x^{-(n-2)(k+1)})-kx^{\left(n-2)(n+km-1\right)}M_{1}^{⁎}(X,x^{-(n-2)(k+1)})$*.*
(iii) $M_{3}(X_{k}^{--},x)=\bar{M_{3}}(X,x^{\left(k+1\right)})+kmx^{\left|km+1\right|}M_{0}(X,x^{\left(k+1\right)})-kx^{\left|km+1\right|}\times M_{1}^{⁎}(X,x^{\left(k+1\right)})$*.*

Corollary 14

(i) $M_{1}(\bar{X_{k}^{--}},x)=M_{1}(X,x^{\left(k+1\right)})+kx^{\left(km+1\right)}M_{1}^{⁎}(X,x^{\left(k+1\right)})+\left(\begin{array}{c} km\\ 2 \end{array}\right)x^{2(km+1)}$*.*
(ii) $M_{2}(\bar{X_{k}^{--}},x)=M_{2}(X,x^{{\left(k+1\right)}^{2}})+kM_{1}^{⁎}(X,x^{\left(k+1)(km+1\right)})+\left(\begin{array}{c} km\\ 2 \end{array}\right)x^{{\left(km+1\right)}^{2}}$*.*
(iii) $M_{3}(\bar{X_{k}^{--}},x)=M_{3}(X,x^{\left(k+1\right)})+kx^{\left|km+1\right|}M_{1}^{⁎}(X,x^{\left(k+1\right)})+\left(\begin{array}{c} km\\ 2 \end{array}\right)$*.*

Corollary 15

(i) ${\bar{M}}_{1}(X_{k}^{--},x)=x^{2(n+km-1)}M_{1}(X,x^{-(k+1)})+kx^{\left(2n+km-3\right)}M_{1}^{⁎}(X,x^{-(k+1)})+\left(\begin{array}{c} km\\ 2 \end{array}\right)x^{2(n-2)}$*.*
(ii) ${\bar{M}}_{2}(X_{k}^{--},x)=M_{-\frac{\left(n+km-1\right)}{\left(k+1\right)},-\frac{\left(n+km-1\right)}{\left(k+1\right)}}^{\prime }(X,x^{{\left(k+1\right)}^{2}})+kx^{\left(n-2)(n+km-1\right)}\times M_{1}^{⁎}(X,x^{-(n-2)(k+1)})+\left(\begin{array}{c} km\\ 2 \end{array}\right)x^{{\left(n-2\right)}^{2}}$*.*
(iii) ${\bar{M}}_{3}(X_{k}^{--},x)=M_{3}(X,x^{\left(k+1\right)})+kx^{\left|km+1\right|}M_{1}^{⁎}(X,x^{\left(k+1\right)})+\left(\begin{array}{c} km\\ 2 \end{array}\right)$*.*

Corollary 16

(i) ${\bar{M}}_{1}(\bar{X_{k}^{--}},x)=\bar{M_{1}}(X,x^{\left(k+1\right)})+kmx^{\left(km+1\right)}M_{0}(X,x^{\left(k+1\right)})-kx^{\left(km+1\right)}\times M_{1}^{⁎}(X,x^{\left(k+1\right)})$*.*
(ii) ${\bar{M}}_{2}(\bar{X_{k}^{--}},x)=\bar{M_{2}}(X,x^{{\left(k+1\right)}^{2}})+kmM_{0}(X,x^{\left(k+1)(km+1\right)})-kM_{1}^{⁎}(X,x^{\left(k+1)(km+1\right)})$*.*
(iii) ${\bar{M}}_{3}(\bar{X_{k}^{--}},x)=M_{3}(X,x^{\left(k+1\right)})+kmx^{\left|km+1\right|}M_{0}(X,x^{\left(k+1\right)})-kx^{\left|km+1\right|}M_{1}^{⁎}(X,x^{\left(k+1\right)})$*.*

## 8 Conclusion

In this paper, we present some explicit expressions for the *F*-index (and co-index) of the *k*th generalized transformation graphs of a molecular graph in terms of various graph invariants. Also, some figures are constructed to show their changes under different *k*th generalized transformation graphs. The Zagreb polynomials, its co-polynomials and their complements are determined for the same transformation graphs. This work will help researchers working in the field of chemical graph theory that has many applications in chemical engineering. In future, we would like to consider a significant extension of the existing work in the literature for many other topological indices and their corresponding polynomials.

## Declarations

### Author contribution statement

D. Maji: Conceived and designed the analysis; Analyzed and interpreted the data; Contributed analysis tools or data; Wrote the paper.

G. Ghorai: Conceived and designed the analysis; Analyzed and interpreted the data; Contributed analysis tools or data.

### Funding statement

This research did not receive any specific grant from funding agencies in the public, commercial, or not-for-profit sectors.

### Data availability statement

No data was used for the research described in the article.

### Declaration of interests statement

The authors declare no conflict of interest.

### Additional information

No additional information is available for this paper.
